# Variation in the *TAS2R31* bitter taste receptor gene relates to liking for the nonnutritive sweetener Acesulfame-K among children and adults

**DOI:** 10.1038/srep39135

**Published:** 2016-12-14

**Authors:** Nuala Bobowski, Danielle R. Reed, Julie A. Mennella

**Affiliations:** 1 Monell Chemical Senses Center, 3500 Market Street, Philadelphia, PA 19104-3308, USA

## Abstract

The nonnutritive sweetener (NNS) acesulfame potassium (Ace-K) elicits a bitter off-taste that varies among adults due to polymorphisms in a bitter taste receptor gene. Whether polymorphisms affect liking for Ace-K by children, who live in different sensory worlds, is unknown. We examined hedonic response to Ace-K among children compared to adults, and whether response was related to common variants of the *TAS2R31* bitter taste receptor gene and to NNS intake. Children (N = 48) and their mothers (N = 34) rated liking of Ace-K, and mothers reported whether they or their children ever consume NNSs via questionnaire. Participants were genotyped for *TAS2R31* variant sites associated with adult perception of Ace-K (R35W, L162M, A227V, and V240I). Regardless of age, more participants with 1 or no copies than with 2 copies of the *TAS2R31* WMVI haplotype liked Ace-K (p = 0.01). NNS-sweetened products were consumed by 50% and 15% of mothers and children, respectively, with no association between intake and *TAS2R31*. The *TAS2R31* WMVI haplotype was partly responsible for children’s hedonic response to Ace-K, highlighting a potential role for inborn differences in vulnerability to overconsumption of Ace-K-containing products. Currently available methods to measure NNS intake yield crude estimates at best, suggesting self-reports are not reflective of actual intake.

The biological drive to prefer sweetness, a taste with powerful hedonic appeal especially among children, confers an advantage in environments of scarcity; it attracts children to mother’s milk and then to honey and fruits, sources of energy^1^. However, within the context of a food supply that provides sweet-tasting foods and beverages in abundance^2^,^3^, this inborn preference can put children at risk for overconsumption of palatable foods and beverages^4^. Indeed, from the age of 2 years, an American child is more likely to eat a manufactured sweet than a fruit on a given day^5^.

Excessive intake of added sugars is associated with increased risk for a variety of diseases, including dental caries, diabetes, obesity, and cardiovascular disease^6^,^7^,^8^,^9^. Despite an overall decrease in consumption of added sugars by Americans over the past decade^10^, children, who as a group prefer higher concentrations of sucrose than adults^1^,^11^,^12^, continue to consume added sugars at levels well above recommendations^9^,^13^,^14^. Sugar-sweetened beverages are the primary source of added sugars in the child’s diet^13^, with more than 90% of children as young as 3 years ever consuming sugar-sweetened beverages^15^.

Presumably in an effort to decrease total added sugars and caloric content of processed foods and beverages, nonnutritive sweeteners (NNSs), which provide sweetness with few to no calories, have become increasingly prevalent in the food supply^16^,^17^, including in products geared toward children [e.g., flavored drink mixes (Kool-Aid)^18^, breakfast pastries (Pillsbury Toaster Strudel^19^), and medical foods (Pedialyte^20^)]. In part because of its stability under high temperatures and synergistic sweetening effect when combined with other sweeteners^21^, Acesulfame potassium (Ace-K) is one of the most frequently used NNSs. However, in addition to sweetness, Ace-K elicits an objectionable bitter off-taste that varies in intensity across individuals, at least among adults^22^,^23^,^24^,^25^. This variation in perceived bitterness is driven in part by polymorphisms in the *TAS2R31* bitter taste receptor gene, a finding confirmed through both psychophysical testing with adults^24^,^25^,^26^,^27^ and functional *in vitro* assays^25^,^27^. Pronounced age-related differences in genotype-phenotype associations for other bitter taste receptor genes and bitter taste stimuli have been observed^28^,^29^,^30^, so we do not know whether the association between *TAS2R31* and Ace-K is evident among pediatric populations.

Despite the important role of taste in development of food preferences beginning early in life^31^, validated age-appropriate methodologies to measure children’s taste responses are lacking^32^,^33^. The forced-choice, paired-comparison tracking procedure is a reliable and valid method^12^ to assess children’s preference for sweet taste (both sucrose^12^,^28^,^34^,^35^ and sucralose^5^) and salty taste^36^,^37^, and its use over more than a decade has significantly broadened our understanding of relationships between taste and other variables, including taste receptor genotype^28^,^38^, race/ethnicity^12^,^38^, and family history of alcoholism^34^,^39^. However, measuring sweet taste preference using this tracking procedure typically takes ~15 min to complete and requires considerable training of the researcher to administer^12^, highlighting the need for a quick and simple method to accurately measure children’s taste response and to examine potential taste effects on food choice in large scale studies, such as the National Health and Nutrition Examination Survey. The 3-point facial hedonic scale which requires users to select a “super good,” neutral, or “super bad” face^40^ to indicate their hedonic response to a stimulus, is both easy to explain to children and easy for a researcher to administer, and children’s understanding of the method is quick to assess^41^. While the 3-point scale has previously been used to measure broad categorizations of whether children like or dislike foods and beverages^41^,^42^, to our knowledge it has not been used to examine genotype-phenotype associations between taste receptor genes and taste hedonics.

To this end, the objectives of this study were to examine individual differences in hedonic response to the taste of Ace-K among children and their mothers using a simple psychophysical method (the 3-point facial hedonic scale) and to examine whether differences were related to common variants of the *TAS2R31* bitter taste receptor gene and to self-reported NNS intake. In addition to psychophysical ratings of a suprathreshold concentration of Ace-K, participants also rated propylthiouracil (PROP or PTU), which served as a control stimulus for two reasons. First, the association between PROP and *TAS2R38* genotype is a classic example of individual differences in bitter taste sensitivity driven by genetics: individuals with the bitter-sensitive genotype (PP) perceive PROP as significantly more bitter than do heterozygotes (AP) or those with the bitter-insensitive genotype (AA), who perceive little to no bitterness^43^. We observe this genotype-phenotype relationship in children, although heterozygous AP children are even more sensitive than AP adults of the same genotype^29^. Second, if hedonic responses to PROP were as expected (e.g., more participants with the bitter-insensitive than with the bitter-sensitive genotype liked the taste of PROP), these results would in part validate the sensitivity of the 3-point facial hedonic scale to detect genetic effects on variation in taste hedonics in children.

## Results

### Participant Characteristics and Completion of Task

The study population consisted of 48 children and their mothers (n = 34), whose race and ethnicity (63% black, 12% white, 25% other or mixed), family income (23% <$15,000, 24% $15–35,000, 35% $35–75,000, 9% >$75,000, 9% did not complete), and mothers’ education levels (77% graduated high school) reflected the diversity of the metropolitan area of Philadelphia^44^. Four children did not complete testing, which was stopped by the researcher when children appeared unfocused or distracted.

### Age- and Gene-Related Effects

There were no significant differences in hedonic response to Ace-K between children and adults: 35% of children and 32% of adults rated the taste of Ace-K as “super bad”, 25% of children and 36% of adults rated its taste as neutral, and 41% of children and 32% of adults rated its taste as “super good” (χ^2^(2) = 1.08; p = 0.58). Nor were there age-related differences in the distribution of *TAS2R31* haplotypes: similar proportions of children and adults had no (21% children, 29% adults), 1 (64% children, 59% adults) or 2 (15% children, 12% adults) *TAS2R31* WMVI alleles (Yates’s χ^2^(2) = 0.19, p = 0.91). Because of the lack of differences between the groups in haplotype distribution and hedonic ratings, age groups were combined to examine hedonic response by genotype.

**Figure 1:**
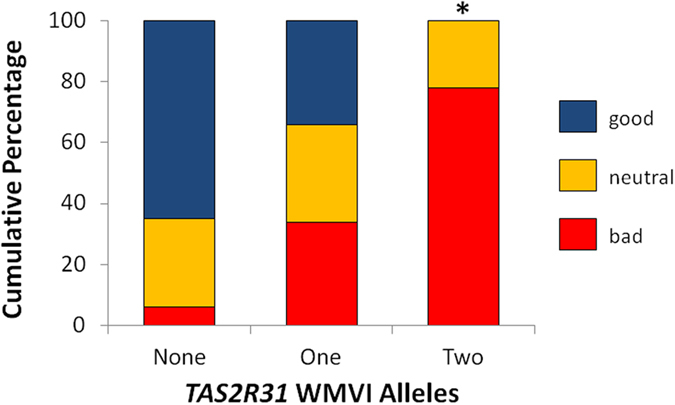
Relation between number of *TAS2R31* WMVI alleles and hedonics of Ace-K. More participants with 2 (n = 9) alleles indicated 0.012 M Ace-K had a bad taste on the 3-point facial hedonic scale than did those with 1 (n = 41) or no (n = 17) alleles (Yates’s χ^2^ = 12.2, p < 0.01).

As shown in Fig. 1, there was an additive effect of the *TAS2R31* gene on Ace-K hedonic ratings: more individuals with no copies (65%) or 1 copy (34%) of the WMVI haplotype liked the taste of Ace-K than did those with 2 copies, none of whom selected the “super good” face to indicate hedonic response on the 3-point scale (Yates’s χ^2^(4) = 12.5, p = 0.01). Partition analyses revealed the significant difference was specifically due to a greater percentage of participants with 2 copies of the WMVI haplotype selecting the “super bad” face compared with those with 1 or no alleles (Yates’s χ^2^(1) = 12.2, p < 0.01).

There were no significant differences in hedonic response to PROP between children and adults: 42% of children and 44% of adults rated the taste of PROP as “super bad”, 49% of children and 47% of adults rated its taste as neutral, and 9% of both children and mothers rated its taste as “super good” (Yates’s χ^2^(2) = 0.16, p = 0.93). Nor were there differences in distribution of *TAS2R38* genotypes by age group: 36% of children and 21% of adults were AA, 42% of children and 50% of adults were AP, and 21% of children and 29% of adults were PP (χ^2^(2) = 1.22, p = 0.54). Because of the lack of differences between the groups in genotype distribution and hedonic ratings, children and adults were combined to examine hedonic response by genotype and to validate the psychophysical method used.

**Figure 2:**
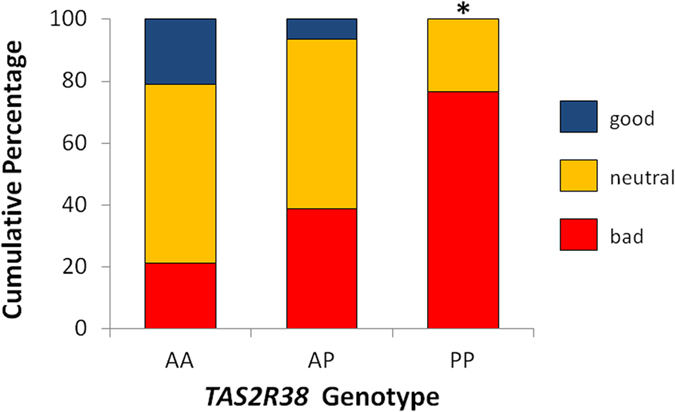
Relation between *TAS2R38* genotype and hedonics of PROP. More participants with the bitter-sensitive PP genotype (n = 17) indicated 56 μM PROP had a bad taste on the 3-point facial hedonic scale than did AP (n = 31) or AA (n = 19) participants (Yates’s χ^2^ = 8.5, p < 0.01).

For the association between PROP and *TAS2R38* genotype, the expected pattern in hedonic response was observed: more AA (21%) and AP (6%) participants liked the taste of PROP than did bitter-sensitive PP (0%) participants (Yates’s χ^2^(4) = 9.79, p = 0.04; Fig. 2). Partition analyses revealed the significant difference was due to a greater percentage of PP than AP or AA participants selecting the “super bad” face to indicate their hedonic response to PROP (Yates’s χ^2^(1) = 8.5, p < 0.01). We also tested for specificity to examine effects of *TAS2R31* on hedonic response to PROP and *TAS2R38* on response to Ace-K, finding no relationships (*TAS2R31* on PROP: Yates’s χ^2^(4) = 0.67, p = 0.95; *TAS2R38* on Ace-K: Yates’s χ^2^(4) = 2.37, p = 0.67).

### NNS Intake

Thirty percent of mothers reported adding NNSs to foods or beverages, and 50% reported consuming diet or sugar-free products. Six percent reported their children used NNSs as a sweetening agent for foods or beverages, and 15% reported their children consumed diet or sugar-free products. Participants were not questioned about specific brands of processed foods and beverages, so it was not possible to assess exactly what NNSs (e.g., aspartame, sucralose, Ace-K) they were consuming. There was no difference in the percentage of mothers or children consuming NNSs by *TAS2R31* genotype (all p’s > 0.13).

## Discussion

Variation in liking for the taste of Ace-K, one of the most prevalent NNSs in the food supply^24^, was significantly associated with variation in the bitter taste receptor *TAS2R31* among both children and adults, with the observed genotype-phenotype association as evident as the association between the *TAS2R38* genotype and liking for the taste of PROP, a classic example of variability in taste sensitivity driven by genetics^43^. For both taste stimuli, the observed results were as expected: participants with the homozygous bitter-sensitive genotypes for *TAS2R31* (two WMVI alleles) and *TAS2R38* (two P alleles) were more likely to dislike the taste of Ace-K and PROP, respectively, than were the others. Such findings both build upon previous reports of associations between polymorphisms of *TAS2R31* and perceived bitterness intensity of Ace-K^24^,^25^, and validate the 3-point facial hedonic scale as an effective measure for assessing a highly subjective psychophysical response.

In the present study, maternal reports indicated that half of mothers and 15% of children had consumed NNS-sweetened products, a finding comparable to National Health and Nutrition Examination Survey estimates for NNS-sweetened beverage intake^45^,^46^. However, several factors suggest a greater percentage of participants in both age groups than reported were likely NNS consumers. First, consumers are generally unable to identify products containing NNSs^47^. Seventy-two percent of parents surveyed on their family’s grocery preferences did not think NNSs were safe for their children to consume, but in a recognition task that required these parents to identify products containing NNSs from a range of processed foods and beverages, 77% of products were not correctly identified^47^. This lack of accuracy occurred despite researchers providing parents with examples of the names of specific NNSs just prior to the task, similar to the methodology used in the present study in which mothers were presented with images of commonly used NNSs while completing a questionnaire on intake. Second, the average consumer is unaware of their own NNS intake. Data from a study of lactating mothers who donated breast milk to be analyzed for the presence of NNSs (Ace-K, saccharin, and sucralose) revealed that of the samples donated by women who reported no NNS consumption, 67% contained Ace-K^48^. This general lack of knowledge regarding NNSs in one’s own diet is further complicated by national food packaging regulations. Unlike nutritive sugars, the amount of NNSs added to foods and beverages is not required on the Nutrition Facts label of processed products, making it difficult to quantify an individual’s intake of Ace-K or any other NNS with any method, and impossible with self-reports collected through traditional methods such as dietary recall or food frequency questionnaires. Though examining whether variation in *TAS2R31* WMVI haplotype is associated with differential consumption of Ace-K–sweetened foods and beverages is an important area for future research, the use of dietary intake records limits the ability to determine whether inborn individual differences have any impact on NNS intake. The unreliability of consumption estimates^16^ may necessitate research to establish whether NNS intake can be estimated from analysis of biological fluids such as blood or saliva, as has been done with breast milk^48^,^49^.

Whether there is a nutritional benefit from consuming NNSs among non-diabetic children is the focus of many commentaries and research articles^50^,^51^,^52^. For example, concern has been expressed over potential negative consequences of regular NNS consumption, including an association between NNS intake and overall poorer diet quality^53^; alteration to composition of gut microbiota^54^; elevations in sweet taste preferences as a result of persistent exposure to the sweet taste of NNS^51^,^55^; and a disruption to flavor-nutrient learning wherein children who learn that sweet taste can be present with few to no calories become at risk for overconsuming sweet taste when present as a high-calorie source of energy, such as sucrose^56^. These concerns may be of particular consequence in the coming years given recent FDA regulations requiring inclusion of added sugars on Nutrition Facts labels of foods and beverages beginning in 2018^57^, leading some to suggest that use of NNSs could increase further as a potential strategy to maintain sweetness but limit added sugar content in the product and consequently on the packaging label^58^,^59^.

Though the focus of the present study was on the genotype-phenotype association between *TAS2R31* and Ace-K, our findings highlight a need for continued research to understand inborn factors that may drive individual differences in intake of ‘sweetness’, potentially making some children more vulnerable than others to overconsumption of both nutritive and NNS-sweetened products^5^. Additionally, in agreement with previous reports^3^,^16^, our findings reinforce a need for effective educational strategies to increase consumer knowledge and literacy regarding NNSs in foods and beverages, and for the modification of food labeling regulations to increase transparency^16^,^60^. Such information is needed to assess whether the changing food supply increases intake of NNSs during the earliest stages of development, from pregnancy^61^, to lactation^44^,^45^, to childhood^16^, and whether early NNS exposure impacts sweet taste preferences and health.

## Methods

For this cross-sectional study, we recruited mothers (N = 34) and their children (N = 48; 6 to 14 years of age; 31 girls, 17 boys) in the Philadelphia area from a list of past participants who asked to be notified of future studies at the Monell Chemical Senses Center. Mothers who were pregnant along with participants with food allergies or taking medication known to affect taste sensitivity were excluded. All procedures were approved by the Office of Regulatory Affairs at the University of Pennsylvania and methods were carried out in accordance with relevant guidelines and regulations. Written informed consent was obtained from each mother, and written informed assent from each child 7 or more years of age. Demographic data were collected by maternal interview. This trial was registered at clinicaltrials.gov as NCT02646956 on December 18^th^, 2015.

### Psychophysical Testing

The study was designed to examine hedonic response to suprathreshold concentrations of a food-grade (Spectrum Chemical, New Brunswick, NJ) nutritive sweetener (sucrose), NNSs (Ace-K, sucralose, aspartame), and other taste stimuli (potassium chloride and PROP) among children and adults over a 2-day period. We present herein the data on 0.012 M Ace-K, a concentration matched to the sweetness intensity of 1.05 M sucrose^62^, which represents the upper limit of children’s sweet preference^12^, and on 56 μM PROP, a concentration which distinguishes individuals based on *TAS2R38* genotype^29^.

Procedures were identical for children and adults, who were tested individually in a private, comfortable room specifically designed for sensory testing. All participants were asked to consume no food or drink other than water for at least 1 h before testing and were acclimated to the testing room for ~15 min. Prior to testing, participants were familiarized with the 3-point facial hedonic scale, made up of faces ranging in emotion from a frowning face (“super bad”) to a neutral face, to a smiling face (“super good”) using Compusense software (version 4.6, Compusense Inc., Guelph, Ontario, Canada). Participants were instructed to select the “super bad” face if they disliked the taste of the solution, the neutral face if they neither liked nor disliked its taste, and the “super good” face if they liked its taste. We assessed whether children understood the task by asking them what face they would point to if they were eating their favorite or least favorite food, as reported to investigators.

In randomized and counterbalanced order, participants were presented with 5 ml of each taste stimulus except PROP in 30-ml plastic medicine cups; PROP was always presented in the last tasting position to avoid carryover effects among participants sensitive to its bitter taste. Participants were instructed to swish the solution in their mouth for 5 s, to expectorate, and to rate the solution by selecting the face on the scale that best represented their hedonic response. A 1-min interval separated each tasting, during which participants rinsed their mouth twice with water.

### Taste Receptor Genotyping

**Figure 3:**
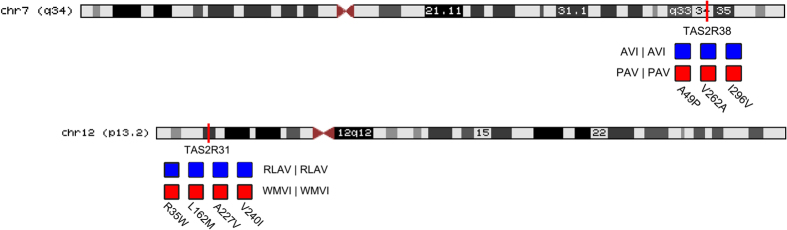
Variants of the bitter receptor genes *TAS2R31* and *TAS2R38*. Boxes denote variant sites, e.g., TAS2R31 R35W indicates the location in the peptide (position 35) where arginine (R) is replaced by tryptophan (W). Non- or less functional genotypes are in blue whereas functional genotypes are in red.

Participants provided DNA samples extracted from saliva (BuccalAmp, Epicenter, Madison, WI, or Genotek, Kanata, Canada), which were used as template in Taqman assays (Applied Biosystems, Foster City, CA) and assayed in duplicate using previously established methods^35^. Though all children provided saliva samples, 14 were excluded from genetic analyses to ensure only 1 sibling per family was studied. All participants were genotyped for several *TAS2R31* variant sites, including those previously shown to be related to perceived bitterness intensity of Ace-K among adults (R35W, L162M, A227V and V240I)^25^. Haplotypes were constructed by tracing the parental origins of the alleles and participants were placed in 1 of 3 groups based on the 2 common diplotypes (RLAV vs WMVI). We also typed the A49P position of the bitter receptor gene *TAS2R38* to verify that we could detect known genotype-phenotype relationships using these methods and in this particular group of participants. Figure 3 illustrates the location of the *TAS2R31* and *TAS2R38* genes, the variants assayed, and the major genotypes of each.

### Questionnaire

Mothers completed an 18-item questionnaire made up of yes/no questions about whether they ever added sugar or NNSs to foods and/or beverages (e.g., coffee, tea) and whether they ever consumed diet or sugar-free foods such as sodas, candy and other sweets, jams/jellies, yogurt, pudding, gelatin, or ice cream. Mothers then answered the same 18 questions for their children; they were provided with pictures of commonly used tabletop NNSs (e.g., Equal, Splenda, Truvia, Sweet’N Low) to assist with recollection.

### Statistical Analyses

For Ace-K and PROP, we determined the percentage of children (N = 33) and adults (N = 34) who selected the “super good,” neutral, or “super bad” face on the 3-point facial hedonic scale. Chi-square analyses were used to examine if hedonic response to either stimulus differed between age groups; to determine if response to Ace-K and PROP differed by *TAS2R31* haplotype and *TAS2R38* genotype, respectively; and to determine whether there was any difference by mother’s *TAS2R31* genotype in reported NNS intake and consumption of diet and sugar-free foods and beverages among both mothers and their children. Yates’s chi-square was used if fewer than 5 participants selected a particular face on the scale for each analysis. When any significant difference was found, follow-up partition analyses were used to further examine the effects and determine where the difference occurred^63^.

## Additional Information

**How to cite this article**: Bobowski, N. *et al*. Variation in the *TAS2R31* bitter taste receptor gene relates to liking for the nonnutritive sweetener Acesulfame-K among children and adults. *Sci. Rep.* **6**, 39135; doi: 10.1038/srep39135 (2016).

**Publisher's note:** Springer Nature remains neutral with regard to jurisdictional claims in published maps and institutional affiliations.
